# Identifying Patient Populations in Texts Describing Drug Approvals Through Deep Learning–Based Information Extraction: Development of a Natural Language Processing Algorithm

**DOI:** 10.2196/44876

**Published:** 2023-06-22

**Authors:** Aline Gendrin, Leonidas Souliotis, James Loudon-Griffiths, Ravisha Aggarwal, Daniel Amoako, Gregory Desouza, Sashka Dimitrievska, Paul Metcalfe, Emilie Louvet, Harpreet Sahni

**Affiliations:** 1 AstraZeneca Cambridge United Kingdom; 2 AstraZeneca Bangalore India; 3 AstraZeneca Wilmington, DE United States; 4 AstraZeneca Gaithersburg, MD United States

**Keywords:** algorithm, artificial intelligence, BERT, cancer, classification, data extraction, data mining, deep-learning, development, drug approval, free text, information retrieval, line of therapy, machine learning, natural language processing, NLP, oncology, pharmaceutic, pharmacology, pharmacy, stage of cancer, text extraction, text mining, unstructured data

## Abstract

**Background:**

New drug treatments are regularly approved, and it is challenging to remain up-to-date in this rapidly changing environment. Fast and accurate visualization is important to allow a global understanding of the drug market. Automation of this information extraction provides a helpful starting point for the subject matter expert, helps to mitigate human errors, and saves time.

**Objective:**

We aimed to semiautomate disease population extraction from the free text of oncology drug approval descriptions from the BioMedTracker database for 6 selected drug targets. More specifically, we intended to extract (1) line of therapy, (2) stage of cancer of the patient population described in the approval, and (3) the clinical trials that provide evidence for the approval. We aimed to use these results in downstream applications, aiding the searchability of relevant content against related drug project sources.

**Methods:**

We fine-tuned a state-of-the-art deep learning model, Bidirectional Encoder Representations from Transformers, for each of the 3 desired outputs. We independently applied rule-based text mining approaches. We compared the performances of deep learning and rule-based approaches and selected the best method, which was then applied to new entries. The results were manually curated by a subject matter expert and then used to train new models.

**Results:**

The training data set is currently small (433 entries) and will enlarge over time when new approval descriptions become available or if a choice is made to take another drug target into account. The deep learning models achieved 61% and 56% 5-fold cross-validated accuracies for line of therapy and stage of cancer, respectively, which were treated as classification tasks. Trial identification is treated as a named entity recognition task, and the 5-fold cross-validated *F*_1_-score is currently 87%. Although the scores of the classification tasks could seem low, the models comprise 5 classes each, and such scores are a marked improvement when compared to random classification. Moreover, we expect improved performance as the input data set grows, since deep learning models need to be trained on a large enough amount of data to be able to learn the task they are taught. The rule-based approach achieved 60% and 74% 5-fold cross-validated accuracies for line of therapy and stage of cancer, respectively. No attempt was made to define a rule-based approach for trial identification.

**Conclusions:**

We developed a natural language processing algorithm that is currently assisting subject matter experts in disease population extraction, which supports health authority approvals. This algorithm achieves semiautomation, enabling subject matter experts to leverage the results for deeper analysis and to accelerate information retrieval in a crowded clinical environment such as oncology.

## Introduction

Recent developments in deep learning–based [1,2] natural language processing (NLP) have enabled transfer learning [3] to be used in automated or semiautomated information extraction using data sets as small as thousands or even sometimes hundreds of entries [4]. While a data set containing billions of words (the full Wikipedia and BooksCorpus content) is necessary to train models such as Bidirectional Encoder Representations from Transformers (BERT) [1], a state-of-the-art deep learning NLP model, fine-tuning this model can be successfully applied to much smaller data sets [4]. Small input data sets are often encountered in practice, and such methods allow applicability to a larger number of problems. Moreover, BERT has demonstrated state-of-the-art performances on a wide variety of tasks, including binary and multiclass classification on balanced and unbalanced data sets or question-answering data sets [1]. When data drift has to be expected, such stability is a strong differentiator.

Besides the fine-tuned BERT deep learning model, we develop a fit-for-purpose rule-based approach. We then compare results of both approaches, and the algorithm that performs best is applied to new data. The results are sent for review and curation to subject matter experts.

In the case study presented in this paper, the goal was to categorize and extract entities from descriptions of drug approvals that would allow us to link a particular patient population and clinical trials to a specific drug approval event. This linkage supports our aim of streamlining information extraction and aiding visualization of the competitive drug approval landscape.

We selected 6 drug targets of relevance to AstraZeneca’s Oncology portfolio and investigate the capability of NLP tools to extract an overview of the competitive landscape for these drug targets. The aim was to retrieve information defining the patient profile—specifically the approved line of therapy and stage of cancer—and references to the clinical trial or trials that support each drug approval.

Machine-learning and rule-based approaches, or their combination, have been used to extract cancer stage automatically from electronic medical records.

Shivade et al [5] and Meng et al [6] have reviewed automatic systems, rule- or machine-learning–based, applied to automatically identify patient phenotype, including but not limited to cancer stage and line of therapy.

A carefully crafted sequence of rule-based approaches and machine learning algorithms allowed cancer stage identification in McCowen et al’s [7] and Yim et al’s [8] studies. In Nguyen et al’s [9] study, a rule-based algorithm was compared to a machine learning approach based on support vector machine, and performances are found to be equivalent. A recent example is described by Hu et al [10], where fine-tuned BERT models were used to identify 14 different named entities and relations among entities. These are then fed to a rule-based postprocessing workflow that answers a list of 22 questions indicative of cancer stage. Most recently, CancerBERT [11] is a fine-tuned BERT-based deep learning model trained to extract 10 types of named entity recognition (NER) entities, including cancer stage.

**Figure 1:**
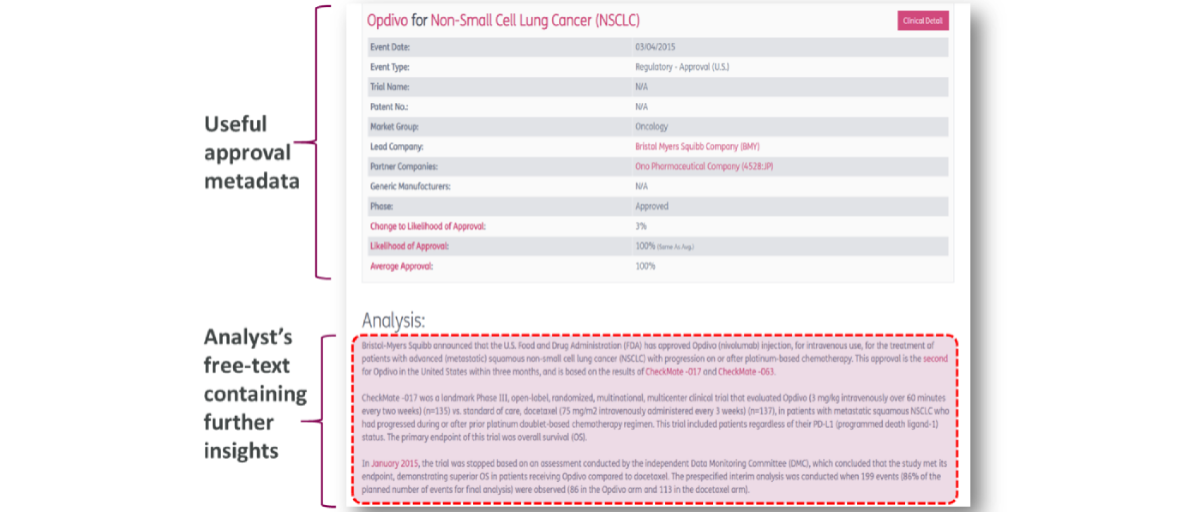
Example approval description from BioMedTracker [18], the data source for this project. The BioMedTracker [18] database contains a repository of standardized drug approval events, reported across several indications and markets. Each event has a number of structured metadata associated with it (eg, disease, approval date, and approval region), as shown in the top half of this figure. Information relating to a more granular description of the patient population is constrained to the unstructured free-text section that is written by an analyst, shown in the lower half of this figure. Texts describing approvals are accessed programatically using a Representative State Transfer application programming interface query (REST API). Image reused with permission by Informa Pharma Intelligence.

Example applications of rule-based approaches used to extract line of therapy automatically are described previously [12-15]. In these studies, cancer stage and line of therapy are often expressed through several indicators that need to be identified individually and then combined. The nature of the documents in our case is less detailed, and a new methodology is needed. A single paragraph of text is available, which sometimes consists of 2 or 3 lines of text only, sometimes more (Figure 1). Stage can be mentioned explicitly, or information can be provided indirectly through words such as “advanced” or “metastatic.” A text describing an approval can cover only 1 cancer stage or a wide range of stages. As for line of therapy, a previous treatment or intervention (resection…) is sometimes mentioned, which helps narrow down the possibilities.

Finally, automatic information extraction of clinical trial characteristics has also been published using carefully crafted combinations of machine learning and rule-based approaches. The extracted information includes trial names as well as relevant information about patient populations enrolled in the trial [16,17]. In our case, we identified, among several trial names, those that lead to compound approval, hence the need for a specially crafted model.

## Methods

### Data Set and Labeling Process

The BioMedTracker [18] database contains a repository of standardized drug approval events, reported across a number of indications and markets. Each event has a number of structured metadata associated with it (eg, disease, approval date, and approval region), as shown in the table in the top half of Figure 1. However, information relating to a more granular description of the patient population (including line of therapy and stage of disease) and any supportive clinical trial is constrained to the unstructured free-text section that is written by an analyst. This can be seen in the lower half of Figure 1. Texts describing drug approvals of interest were accessed programatically from the database using a Representative State Transfer application programing interface (API) query.

We focused on approval events in 6 drug targets, which were included sequentially as the project evolved. The drug targets taken into account were (1) EGFR (Epidermal Growth Factor Receptor), (2) human epidermal growth factor receptor 2/neu or ErbB-2, (3) Cytotoxic T-Lymphocyte Antigen 4, (4) Programed death-1 receptor/Programed death ligands (1 and 2), (5) Poly ADP-Ribose Polymerase, and (6) Bruton’s Tyrosine Kinase, which are all of relevance to AstraZeneca’s drug portfolio.

In terms of preprocessing, hyperlinks were deleted from input texts. Information about line of therapy and cancer stage was found in the first 2 paragraphs of text, so only these were considered for these tasks. The full text was used to identify trials leading to an approval.

A manual labeling process was applied to ensure consistency; 2 subject matter experts split the task of labeling 433 texts describing approvals, while an independent third labeler reviewed their work to ensure accuracy and consistency. The task is difficult as line of therapy and cancer stage are sometimes described indirectly. We used Label-studio [19] to perform the labeling task.

We found that both line of therapy and cancer stage showed a large number of possible classes in the data (Table 1), and for the purposes of model training, pooled some of these categories together to make the classification task more manageable.

The final list of refined classes was selected based on their frequency and an assessment of how useful an individual class would be to the project, as judged by a subject matter expert; this information was also used to assign an ordinal rank to each class, lower ranks corresponding to more common classes.

To map from the initial list to the final list, we developed a binning algorithm that chooses the training class with the highest rank that overlaps with the labeled class. For example, in Table 2, “Second line” was ranked third and “First line” was ranked fourth; therefore, a labeled class with the categories “First line; Second line; Third line” would have the training class value of “Second line.” The highest-ranked class was always assigned the null set. This functioned as the default value for when there is no overlapping training class in the labeled class.

Algorithmically, this means the following:

base_features = {(i,j,k), (i,k,l), (m,n), (k), …},

target_classes_reverse_order = {(i), (j,k), (j), (k), …}

text_target_class = null

for text in texts:

for base_feature in base_features:

for target_class in target_classes_reverse_order:

if base_feature in target_class:

text_target_class = target_class

**Table 1:** Labeled classes present in the data set for line of therapy and stage of cancer after labeling. These classes are input into the binning algorithm to produce the training classes seen in Table 2.

Class			Texts describing approvals, n
**Line of therapy**			
	First line	123	
	Second line	114	
	*blank*	62	
	Maintenance and Consolidation	45	
	First line; Second line	19	
	Second line; Third line	18	
	Fourth line or Greater; Third line	17	
	Adjuvant	14	
	Third line	5	
	Fourth line or Greater; Second line; Third line	5	
	Fourth line or Greater	3	
	Maintenance and Consolidation; Third line	3	
	First line; Second line; Third line	3	
	Adjuvant; Second line; Third line	2	
**Stage of cancer**			
	Stage III; Stage IV	176	
	Stage IV	72	
	*blank*	50	
	Relapsed	41	
	Relapsed; Stage III; Stage IV	40	
	Stage III	19	
	Relapsed; Stage IV	16	
	Extensive stage	11	
	Stage I; Stage II; Stage III	3	
	Stage I	3	
	Stage I; Stage II	2	

**Table 2:** Training classes used for line of therapy and stage of cancer derived by the binning algorithm and ordered by input rank.

Class		Texts describing approvals, n
**Line of therapy**		
	*Blank *	45
	Maintenance/Consolidation	79
	Second line	163
	First line	123
	Third line	23
**Stage of cancer**		
	*Blank*	41
	Stage III; Stage IV	201
	Stage IV	73
	Relapsed	61
	Relapsed; Stage III; Stage IV	57

### NLP Algorithm Development

Off-the-shelf packages are available that return state-of-the-art results on many different benchmarking data sets. Here, we use the transformers library from huggingface [20,21].

We applied transfer learning [2] and fine-tuned a DistilBERT [22] model, a distilled version of BERT that runs faster while retaining comparable performance. We attempted to use BioBERT [4] because models adapted to medical literature have been shown to increase scores [23]; however, here, performance did not improve. Along these lines, we also fine-tuned a domain-adapted BERT-based model, using trial titles from the Trialtrove database as text and patient population categories that had been tagged by a Trialtrove analyst as the target. The performance of this model was disappointing, and we concluded that syntaxes were too different between Trialtrove titles and BioMedTracker approval descriptions, possibly because titles are too short to allow the model to learn.

Line of therapy and stage of cancer extraction are treated as classification problems, while trial identification is treated as a NER task. Preimplemented flows are available in the Hugging Face library [24,25] and we adapted them to our needs. Selecting only the first 2 paragraphs of text led to better results in the classification tasks, while using the full text was found to be best for the NER task, probably because information relating to the line of therapy or stage of cancer is located at the beginning of the text, while trials leading to approval can be found either at the beginning, or toward the end. We also deleted HTML tags, which generally correspond to hyperlinks leading to trial description.

As a benchmark, we developed a rule-based text mining approach on the same data. Subject matter experts gathered common examples of words and phrases that were associated with their choice of line of therapy or stage of cancer. These examples were used as a lookup list in the text-mining model.

The accuracy of the rule-based approach was then calculated and compared with the cross-validated accuracy from the deep learning approach. The highest score was considered as the winning model. Predictions using this winning model were used to prepopulate label-studio input to guide the labeling process when new texts describing approvals became available or new drug targets were taken into account.

### Ethical Considerations

This study is exempt from human subjects’ research review as no human subjects were involved.

## Results

### Classification Tasks: Line and Stage

We observed the following results for the classification task.

For the BERT-based models, we display 5-fold cross-validated accuracy, defined in recent literature as the best available metric for classification [1,26]. This means that the data set is divided into 5 segments, which are successively considered as test data sets; we train the model on 4 segments, that is, 80% of the data, and test it on the remaining segment, that is, 20% of the data. Then the next data segment is considered as test data set. Finally, the 5 results are averaged.

We followed the methodology proposed in appendix A.3 of Devlin et al [1] and performed a grid search over batch size (possible values: 16 and 32), learning rate (possible values: 2e-5, 3e-5, and 5e-5), and number of epochs (possible values: 2, 3, and 4). Devlin et al [1] report in appendix A.3 that searching over these hyperparameters worked well across all tasks they worked on, which include binary and multiclass classifications, balanced and unbalanced data sets, and question answering tasks.

**Figure 2:**
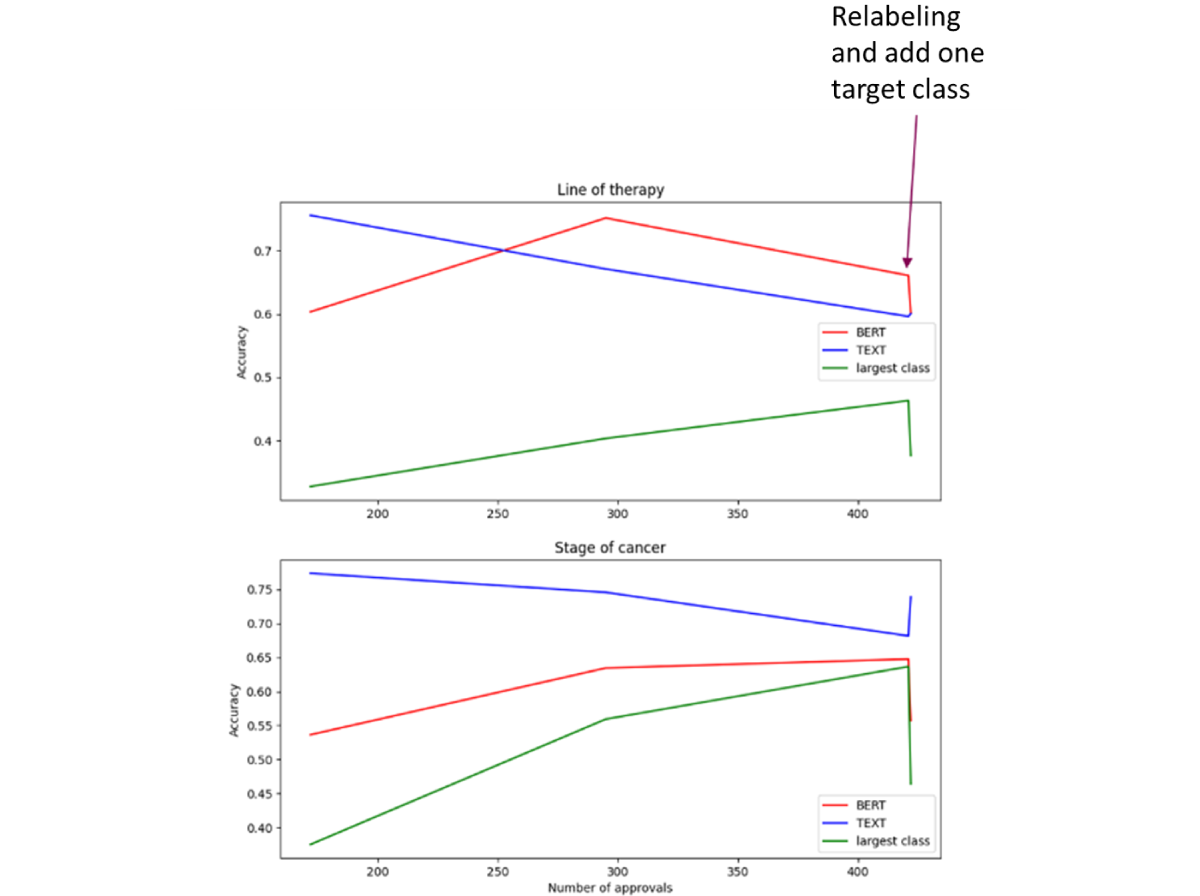
Application to the BioMedTracker data set, performance of the fine-tuned bidirectional encoder representations from transformers (BERT) models at key stages for text classification corresponding to “Line of therapy” and “Stage of cancer.” Rule-based text mining (TEXT) accuracy decreases when more texts describing approvals are taken into account, as line of therapy or stage of cancer is expressed with slightly different formulations from one approval to the next. Deep learning accuracies generally increase when more texts describing approvals are added. Marked changes appear on the right-hand side of the curve, following expert’s intervention to homogenize the labeling and the addition of one target class.

5-fold cross-validated accuracies (red line in Figure 2) generally increase with the number of texts describing approvals, similarly to benchmark data sets (Multimedia Appendix 1 [27-43] ). As a second observation, we notice some local decreases, similar to those observed for benchmark data sets, for example, TREC-6 or IMDB, for similar abscissas (Multimedia Appendix 1). Based on these 2 observations, benchmark data sets provide a good understanding of the fine-tuned BERT models’ behavior.

Due to the unbalanced data set, we also displayed the percentage of inputs from the largest class (green line). This percentage fluctuates through time as new drug targets are included sequentially. A simple algorithm that would place all entries in the largest class would return a score corresponding to the green curve. As an example, for stage of cancer, the proportion of the class corresponding to “stage III; stage IV” reached almost 65% during the project.

For the rule-based text mining model (blue line in Figure 2), accuracies decreased as new texts describing approvals were added to the database. This is in line with expectations: the dictionary of expressions was established early on and not modified, and new texts describing approvals can only bring more diversity in the expressions.

Marked changes at the end of the curves correspond to two key operational decisions: (1) labeling was refined and homogenized and (2) the number of different classes considered in the classification tasks was increased from 4 to 5 (all 5 lines from Table 2 for line of therapy and stage of cancer were taken into account instead of the first 4 lines only).

Current scores of 5-fold cross-validated accuracy were 61% for line of therapy with the fine-tuned BERT model and 60% for the rule-based approach. For cancer stage, they were 56% and 74%, respectively. These scores are displayed in Table 3. Although these scores may seem low, it is important to understand that each model performs multiclass classification with 5 classes each, so they are doing much better than random.

**Table 3:** Current 5-fold cross-validated accuracy scores are reported for line of therapy and cancer stage classification classes, and 5-fold cross-validated *F*_1_-scores are reported for the Clinical Trials Named Entity Recognition task.

	Line of therapy, %	Cancer Stage, %	Clinical Trial, %
Fine-tuned BERT^a^ model	61	56	87
Rule-based approach	60	74	—^b^

^a^BERT: bidirectional encoder representations from transformers.

^b^Not available.

### Model Interpretability

Model interpretability is key in deep learning algorithms, and models whose results are well understood can be preferred to less interpretable, higher-accuracy models [44]. We use LIME [44] to understand what the classification models see in the data.

**Figure 3:**
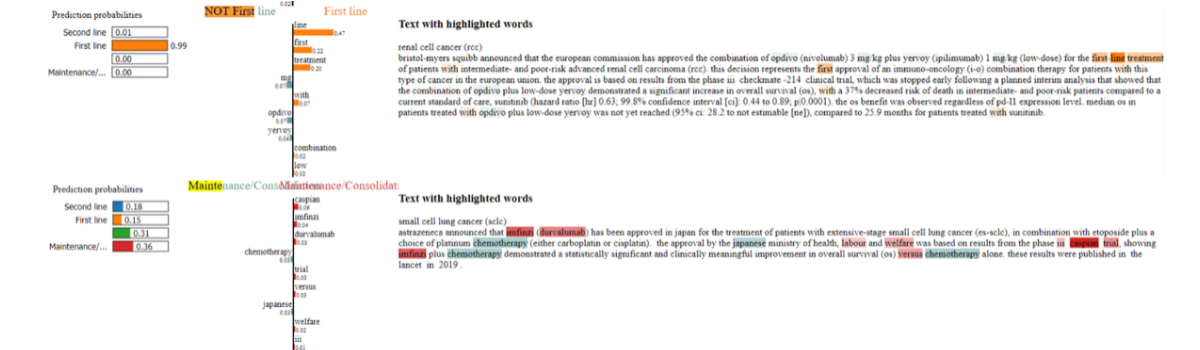
Model interpretability by LIME algorithm. Top: typical LIME results for first line of therapy; bottom: example where the model fails. Left: scores for each category. The highest score corresponds to the model’s results. A highest score close to one is an unambiguous decision, while a lower highest score is a less certain decision. Middle: most important words, where positive values increase the model’s score, and negative values decrease it. Right: input text, important words are highlighted.

LIME results are generally easy to interpret, which builds confidence in the models. For line of therapy, the word “first” appeared repeatedly as the most important word for the class “First line,” and the word “maintenance” appeared often as the most important word for the class “Maintenance/Consolidation.” Figure 3 illustrates this observation on 1 typical example for the class “First line” (top). On the left-hand side of Figure 3, LIME displays scores corresponding to individual classes, and the highest score is the BERT-based model’s result. When the highest score is close to 1, the choice is unambiguous for the model (Figure 3, top, 99% for class First line). In other circumstances, the choice is more balanced (Figure 3, bottom), and in this second example, the model fails to predict the correct class. In the middle part of Figure 3, LIME displays words leading to decision with the most important word at the top and the least important word at the bottom. This ranking was obtained by LIME through deletion of randomly selected words in the text and the reevaluation of the final score. Words appearing with the color of the class reinforce the decision taken by the algorithm, while words displayed in blue weaken the decision. In the right part of Figure 3, LIME displays the input text and highlights the most important words.

Besides these straightforward cases, other words appear as important which are a lot less intuitive. For example, “Korea” was often identified as important in first line and “carcinoma” in second line. These examples show that biases can appear when applied to a new data set. We think that these biases will disappear or be attenuated when the number of inputs increases, something to be checked over time. Since a subject matter expert is involved to correct the results of the algorithm before they are used in the internal software, these possible biases are appropriately handled in the project (see section Deployment to production).

### NER Task: Clinical Study

To identify trials leading to an approval, we adopted a NER algorithm [45]. We selected 5-fold cross-validated *F*_1_-score as a metric for this task, in agreement with recent literature [1,26] for NER tasks. *F*_1_-scores are the harmonic mean of precision and recall and are classically used as a measure of success in NER tasks (unbalanced problems, where accuracy is not sufficient as a metric) [45]. We also applied the hyperparameters grid search strategy described for the classification tasks. In this project, we found that concatenating the BioMedTracker data set with another data set from the literature [46-48] and solving for all end points simultaneously was necessary to get a 5-fold cross-validated *F*_1_-score of 87%, as reported in Table 3. Table 4 illustrates this process and summarizes the number of entities per class available in the merged data set when the merge is done with the wnut data set [48]. The improved scores are in agreement with previous work [49-52], which reports that “multitasking” improves NER results. However, multitask learning generally leads to a few percent increase in *F*_1_-score. In this study, the *F*_1_-score is null when we only use the BioMedTracker data set, and it reaches 87% when we concatenate this data set with one of the conll, ncbi, or wnut data sets [46-48].

**Table 4:** Number of entities per class when the data set is merged with the wnut [48] data set for simultaneous named entity recognition problem resolution. The data set from this study contains only 1 entity type, named clinical trial in the table. All other entity types come from the wnut [48] data set.

Metric	Entries per class in the train data set, n
person	470
location	74
corporation	34
product	114
creative work	104
group	39
clinical trial	345

### Deployment to Production

**Figure 4:**
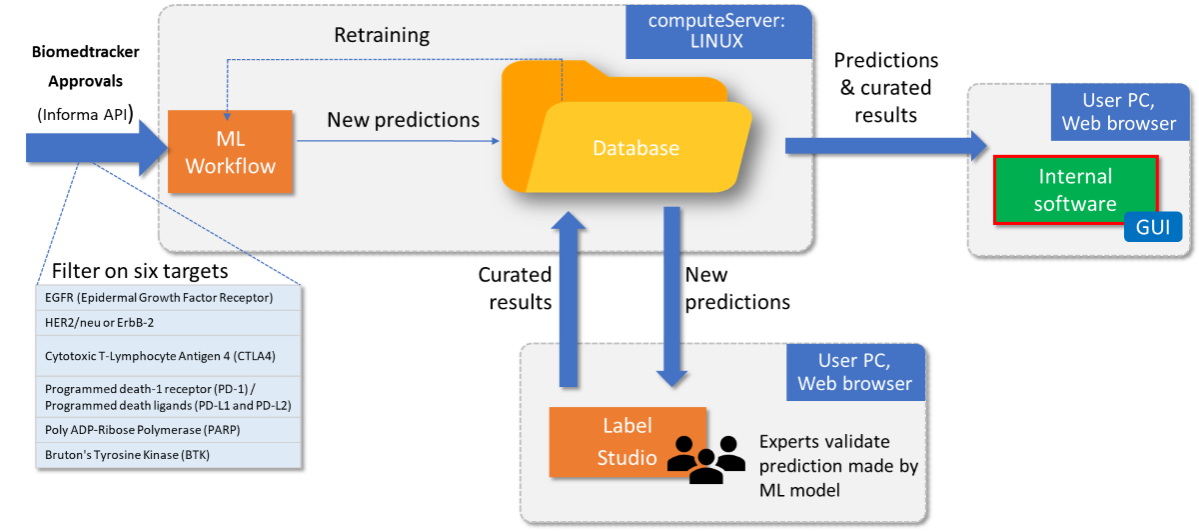
Full workflow as deployed in preproduction phase. New texts describing approvals are collected automatically from the BioMedTracker API [18]. Predicted labels are calculated using both the basic text mining approach and the deep learning approach. The algorithm leading to the highest accuracy is selected. The data set is then released both in the internal custom-made software and to subject matter experts performing the labeling. API: application programming interface; ErbB2: erythroblastic oncogene B; GUI: graphical user interface; HER2: human epidermal growth factor receptor 2; ML: machine learning.

Figure 4 illustrates the deployment to production of the 3 models described above. New texts describing approvals were collected automatically from the BioMedTracker API [18]. Predicted labels were calculated using both the rule-based text mining approach and the deep learning approach described above. The algorithm leading to the highest accuracy was selected, and its results are displayed.

The data set was then released both in internal software and to subject matter experts performing the labeling. Results that correspond to predictions are explicitly flagged as predictions to the user.

## Discussion

### Principal Results

We have developed and put in 3 deep learning models corresponding to fine-tuned versions of the BERT model. Each model is designed to automatically analyze free text describing approvals taken out of the BioMedTracker database and answer one of the following questions: (1) Which line of therapy has the compound been approved for? (2) Which stage of cancer has the compound been approved for? (3) Which clinical trials have supported this approved indication? The first 2 questions have been addressed as classification tasks, while the third question was addressed as an NER task. For this purpose, we have used publicly available packages that allow fine-tuning the BERT model with relative ease [24,25], and we have used published grid search strategies for the hyperparameters [1].

Current scores of 5-fold cross-validated accuracy were 61% and 56% for line of therapy and cancer stage, respectively, and 87% 5-fold cross-validated *F*_1_-scores for clinical trial. We have compared a rule-based approach for line of therapy and cancer stage, whose current scores are 60% and 74%, respectively.

The tasks described in this paper are challenging because they rely on a variety of subtly different text formulations. Hence, machine learning results help focus the analysis of the subject matter expert. For example, they help identify quickly unambiguous cases (top of Figure 3): the model scores high (99% for class “First line”), and the highlighted words indicate the reason for the decision (the words “first-line treatment” are highlighted in the text). The second example at the bottom of Figure 3 is more ambiguous, and the subject matter expert can focus on the analysis. Overall, the 3 machine learning models enable subject matter experts to leverage the results for deeper analysis and to accelerate information retrieval in a crowded clinical environment such as oncology.

### Limitations

The main limitation of the application of deep learning to the BioMedTracker data set is the size of the labeled training data set, which currently is equal to 433 texts describing approvals. More training instances will become available when additional drug targets are considered or when new approval descriptions will be stored in the BioMedTracker database.

It also seems that our problem can be considered a complex problem if we take as a comparison point data sets from the literature used in Multimedia Appendix 1. Indeed, when we add more entry texts, accuracies increase slowly, at a rate similar to the Yahoo! Answers data set (40% accuracy with 200 entry texts and 77% accuracy for all 1.4 million texts).

This small number of training instances leads to relatively low scores for the 2 classification tasks: the current 5-fold cross-validated accuracies for line of therapy and stage of cancer are 61% and 56%, respectively. However, these accuracies are still much better than random choice alone because each model comprises 5 different classes.

Mitigation of these low accuracies for downstream, dependent systems is handled by the production pipeline, since a subject matter expert verifies and corrects the automatic labels produced by the deep learning model so as to return reliable results to end users.

Despite the lower accuracies seen for the classification tasks, subject matter experts reported that the labeling experience was improved by the presence of model predictions; even for a human, it is a nontrivial task to assess the approved populations for a large number of event descriptions.

### Comparison With Previous Work

In this work, we address the problem of extracting information for competitive intelligence. NLP tools have been widely applied to extract information from electronic health records [5-15,16,17,53-55]. Even though the targets can be similar, for example, cancer stage or line of therapy, the nature of the documents is different, a lot less detailed in our case, and a new methodology is needed.

### Conclusions

We have described the development and application of 3 deep learning models, fine-tuned from BERT [1]. They aim at extracting structured information from unstructured text, aiding information extraction and visualizations in downstream systems. The first model classifies the text describing the approval (Figure 1) in 1 of 5 categories corresponding to line of therapy. The second model performs the same task for cancer stage. The third model identifies trials in the paragraph only if they lead to the approval. We compared the results of these deep learning models to rule-based approaches for line of therapy and cancer stage.

In our case, although much better than random, accuracies achieved are insufficient for automation, and human intervention is necessary. We describe how we implement human intervention, which leads to a process that is effective for the users, subject matter experts, and machine learning engineers.

Accuracies are expected to improve through time as more training data become available. However, in the meantime, subject matter experts already find these results to be an insightful guide to labeling, saving much-needed time for extracting this information to support clinical insights and decision-making.

## Appendix

Multimedia Appendix 1 Comparison with benchmark datasets.

## Abbreviations

API: application programming interface
BERT: Bidirectional Encoder Representations from Transformers
NER: named entity recognition
NLP: natural language processing

## Data Availability

Data sets used in Multimedia Appendix 1 are publicly available and relevant links are provided. BioMedTracker data are proprietary and are not allowed to be made public. Data for this study may be requested and purchased from BioMedTracker.
