# Supplementary material for: Identifying Patient Populations in Texts Describing Drug Approvals Through Deep Learning–Based Information Extraction: Development of a Natural Language Processing Algorithm
Source: JMIR Form Res. 2023 Jun 22;7:e44876. doi: 10.2196/44876 (PMC10337300; doi:10.2196/44876)
Supplement: Multimedia Appendix 1 [file formative_v7i1e44876_app1.doc]

# Comparison with benchmark datasets

**Benchmarking datasets**

We used benchmarking datasets to visualise what to expect from selected processing workflows [20, 21], when the number of input texts increases. We focused on the most cited benchmarking datasets for both classification [26] and NER [44] which were available in a user friendly format [27].

Overall, we used six benchmarking datasets for text classification:

- The AGNews [28, 29] dataset is made of news article gathered from the web. It counts four classes, each with 30000 articles in the train dataset, and there are 7200 articles in the test dataset.
- The DBPedia dataset [30] is a community effort to extract structured information from Wikipedia and to make this information available. Here we use a subset of DBPedia [31] which focuses on 14 classes, with a training set of 40000 samples per class, and a test set of 5000 samples per class.
- The Text REtrieval Conference (TREC) [32, 33] is a dataset comprising six classes, and 5500 samples.
- The 20news dataset [34, 35], comprises 20 classes of 1000 newsgroup documents each.
- The IMDB dataset [36, 37] comprises movie reviews for sentiment analysis (binary classification). It counts 25000 samples for training, and 25000 for testing.
- The Yahoo! Answers dataset [38, 39] counts 1.4 million entries for training, and 60000 for testing, in 10 classes.

For NER tasks, we gathered four datasets:

- The conll dataset [45, 40] contains sentences where names of persons, organisations, and locations are clearly labelled. It comprises approximately 6000 entities per category for the training set, and 1500 per category for the test set.
- The ncbi dataset [46, 41] contains PubMed abstracts where disease names have been annotated. There are approximately 5000 disease mentions in the training set, 1000 in the development set, and 1000 in the test set.
- The wnut dataset [47, 42] is focused on rare entities. Twitter is the source of data for the training set, but reddit, youtube and stackexchange are also used for the development and test datasets. Person, location, corporation, product, creative-work, group are labelled with between 30 and 500 mentions in the development and test datasets.
- The V CDR dataset [43] contains 1500 pubmed articles, with ~6000 diseases and ~3000 chemicals annotated.

### Comparison with benchmarks

Given the small size of the input dataset, we evaluated reasonable expectations of final accuracies, and the potential increase of the accuracy with increased data availability. To this end, we applied selected processing workflows [24, 25] to selected benchmark datasets [28, 30, 32, 34, 36, 38, 43, 45, 46, 47].

We randomly subset each benchmarking dataset to visualize accuracy improvements as a function of input size (Figure 1). Datasets were split into train and test datasets in proportions 80/20% and scores were evaluated on the test dataset. Accuracy is chosen as a metric in agreement with state-of-the-art literature for a classification task [26, 1]. For display purposes, outputs calculated with the full dataset are shown at an abscissa of 30000 for each dataset, although dataset sizes are very different. Predominantly, these were within 1-2% of state-of-the-art accuracies, with the exception of three datasets: two datasets [34, 36] had a difference of 4%, and a third dataset [38] had a difference of 6%. These curves lead us to a few key observations:

- the appropriate amount of data to reach applicable accuracies largely depends on the problem. For the DBPedia dataset, 200 entries are sufficient to reach almost 100% accuracy, i.e. full automation. For the Yahoo! Answers dataset, 200 entries only lead to 50% accuracy, and even the state-of-the-art accuracy when using all 1.4 million entries is 77% accuracy only, which means that human validation will always be needed.
- for small numbers of entries, the curves are not strictly rising, oscillations are observed. In this part of the curve, adding entries can lead to decreased accuracies. This needs to be made very clear early in the project to manage stakeholders’ expectations.

For the NER task, we followed the methodology proposed in appendix A.3 of [1] and performed a grid search over batch size (16 and 32), learning rate (2e-5, 3e-5, and 5e-5), and number of epochs (2, 3, 4). In [1], appendix A.3, it is explained that these hyperparameters provide good results across all tasks, which comprise binary and multi-label classifications, on balanced and unbalanced datasets, or question answering tasks, among other examples. Indeed, our NER task is an unbalanced binary classification problem. We selected the best result from this grid search, according to the F1 score metric, selected in agreement with recent literature [44, 1] for a NER task. Again, the majority [45, 46, 47] of outputs were within 4-5% of state-of- the-art accuracies, and within 12% in one case [43].


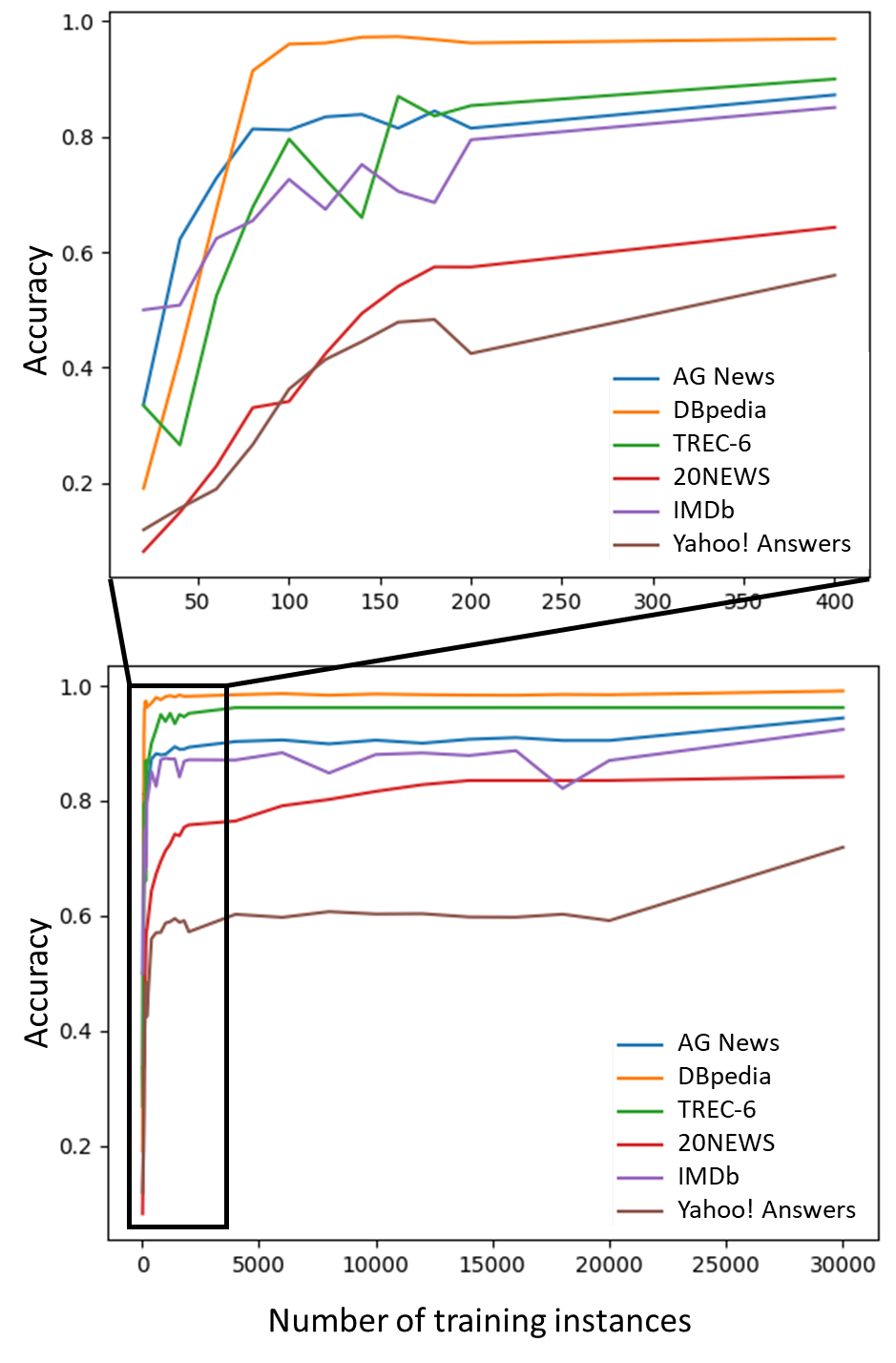


Figure 1: Analysis of benchmark datasets, for text classification. Accuracies generally increase with the number of text entries, for the selected datasets [28, 30, 32, 34, 36, 38]. Some local oscillations are observed for small dataset sizes (between 100 and 400, for TREC-6 and IMDb for example), and lead to local decreases of accuracy. Datasets on this figure have very different sizes: for example, TREC-6 train dataset counts 5400 entries, while Yahoo! Answers train dataset counts 1.4 million entries. For representation purposes, we decided to display accuracies retrieved with the full dataset at an abscissa of 30000 for all 6 datasets.
